# Early loss of T lymphocyte 4-1BB receptor expression is associated with higher short-term mortality in alcoholic hepatitis

**DOI:** 10.1371/journal.pone.0255574

**Published:** 2021-08-05

**Authors:** Lotte Lindgreen Eriksen, Morten Aagaard Nielsen, Tea Lund Laursen, Bent Deleuran, Hendrik Vilstrup, Sidsel Støy

**Affiliations:** 1 Department of Hepatology and Gastroenterology, Aarhus University Hospital, Aarhus, Denmark; 2 Department of Biomedicine, Aarhus University, Aarhus, Denmark; Harvard Medical School, UNITED STATES

## Abstract

**Objectives:**

In alcoholic hepatitis (AH), dysfunctional T lymphocytes may contribute to the high mortality from infections. T lymphocyte activation is governed by the expression of co-stimulatory receptors such as 4-1BB balanced by inhibitory receptors such as Programmed Death receptor 1 (PD-1). 4-1BB expression is unaccounted for in AH, while PD-1 is elevated. We characterized expression of 4-1BB and PD-1 and the associated T lymphocyte functional status in AH and investigated whether these were associated with short-term mortality.

**Methods:**

Thirty-five patients with AH (at diagnosis and days 7 and 90) were compared with healthy controls (HC). Spontaneous and *in vitro* stimulated receptor expression were quantified by flow cytometry, and plasma proteins by ELISA.

**Results:**

At diagnosis, the patients showed increased stimulated 4-1BB responses of CD4^+^ T lymphocytes. Also, the frequencies of PD-1^+^ T lymphocytes both with and without co-expressed 4-1BB were increased. Further, interferon-gamma was predominantly produced in T lymphocytes co-expressing 4-1BB. A decrease in the frequency of spontaneous 4-1BB^+^ T lymphocytes and an increase in soluble 4-1BB during the first week after diagnosis were associated with higher mortality at day 90 in AH. PD-1 expression showed no systematic dynamics related to mortality.

**Conclusions:**

We found an increased stimulated 4-1BB response of T lymphocytes in AH and early loss of these lymphocytes was associated with a higher short-term mortality. This suggests a role of T lymphocyte 4-1BB expression in the progression of AH.

## Introduction

Patients with alcoholic hepatitis have a high susceptibility to infections which carries a high mortality [1, 2]. There is solid evidence for reduced antimicrobial functions in both monocytes and neutrophils, whereby these patients largely rely on their adaptive immune system [3, 4].

4-1BB is a glycosylated, co-stimulatory receptor which is induced on activated T lymphocytes and vital for T-cell receptor (TCR) signal transduction [5, 6]. 4-1BB belongs to the tumour necrosis factor (TNF) receptor superfamily along with other co-stimulatory receptors such as OX40 [7]. Signalling through 4-1BB induces proliferation, cytokine production (e.g. interferon (IFN)-gamma, and survival in T lymphocytes). Therefore, 4-1BB plays an important role in maintaining normal immune function [8–10]. Likewise, animal studies point to an important role of 4-1BB in fighting infections [11, 12]. The 4-1BB ligand (4-1BBL) is primarily expressed on antigen presenting cells and promotes T lymphocyte activation via 4-1BB [13]. 4-1BB can be cleaved from the surface by a disintergrin and metalloprotease (ADAM)-17 leading to the formation of soluble (s) 4-1BB [14]. This soluble form may act as a competitive inhibitor of the binding between 4-1BB and 4-1BBL [15, 16].

Patients with AH often have lymphopenia. Moreover, the inhibitory receptors Programmed Death receptor-1 (PD-1) and T-cell immunoglobulin Mucin-3 (TIM-3) are upregulated on T lymphocytes, and this is associated with reduced antimicrobial functions and a cytokine production dominated by the anti-inflammatory interleukin-10 (IL-10) [17]. Furthermore, galectin (Gal)-9 was found elevated in AH [17]. Interestingly, Gal-9 can bind both 4-1BB and TIM-3. Binding of Gal-9 to 4-1BB potentiates the interaction between 4-1BB and 4-1BBL and thereby increases cytokine production [14, 18–20].

We hypothesized that T lymphocyte 4-1BB expression is increased in AH and associated with better T lymphocyte antimicrobial cytokine production and reduced mortality. We, therefore, characterized the T lymphocyte expression of 4-1BB in relation to PD-1 and Gal-9 and short-term mortality in patients with AH.

## Methods

### Patients and samples

The study was approved by Danish Central Region Committee of health research ethics (1-10-72-40-13) and Datatilsynet (2007-58-0016). All patients provided written, informed consent prior to study inclusion. The study population consisted of 35 patients with AH consecutively recruited from the Department of Hepatology and Gastroenterology, Aarhus University Hospital between March 2013 and March 2018. Patients were included when diagnosed with AH based on the following criteria: a history of excessive alcohol consumption with a period of abstinence of less than 4 weeks prior to disease presentation; acute jaundice with presentation within the previous 2 weeks (serum bilirubin > 80 μmol); age between 18 and 75 years. Exclusion criteria were other underlying liver disease, hepatocellular carcinoma, biliary stones, uncontrolled infection, upper gastrointestinal bleeding, or immunomodulatory therapy within the past 8 weeks. According to local clinical guidelines at the time of study, patients were treated with either pentoxifylline (prior to 2015) or prednisolone when their Glasgow alcoholic hepatitis score (GAHS) was 9 or above. Blood samples were collected at the day of diagnosis prior to initiation of pentoxifylline or prednisolone and at days 7 and 90 after diagnosis and liver biopsies were obtained when possible at diagnosis (n = 21). Peripheral blood mononuclear cells (PBMCs) were isolated from EDTA-whole blood samples by Ficoll-Hypaque (GE Healthcare Biosciences, Uppsala, Sweden) gradient centrifugation and stored until analysis at -140°C. Plasma and serum were obtained from centrifugation of whole blood at 1800g for 10 minutes and stored at -80°C. Blood samples from age- and sex-matched healthy controls were obtained from the Department’s biobank. Healthy liver tissue was collected from the rim of resectates during operations for metastatic colon cancer at the Department of Abdominal Surgery, Aarhus University Hospital [21].

### Multiparameter flow cytometry

At the time of analysis, PBMCs were thawed (viability 80% on average measured with Countess, Invitrogen) and either stained immediately or incubated (2*10^6^ cells/mL) in culture media (RPMI + Penicillin and Streptomycin) with 0.1 ug/mL anti-CD28 (anti-human CD28, BD Pharmingen, cat.number 556620) in wells pre-coated with 0.1 ug/mL anti-CD3 (Orthoclone OKT-3, Ortho Biotech) and incubated for 48 hours at 37°C in a 5% CO_2_ atmosphere. Cells were harvested in PBS on ice.

Cell suspensions were blocked using 0.1 mg human immunoglobulin (Privigen, CSL Behring, Denmark) pr. mL cell suspension and incubated for 15 minutes at 4°C. Afterwards, surface staining was performed on unstimulated and stimulated cells using optimised volumes of the following fluorescent-conjugated monoclonal antibodies: a T lymphocytes panel with anti-CD4-PerCP-Vio700 (Miltenyi, Germany, cat.number 130-113-228), anti-CD8-Pe-Vio770 ((Miltenyi, cat.number 130-110-680), anti-4-1BB-APC (Biolegend, cat.number 309810), anti-Gal9-BV421 (Biolegend cat.number 348920), anti-PD-1-PE (Biolegend, cat.number 329906), anti-TIM3-eFlour-780 (eBioscience, cat.number 47-3109-42) and anti-OX40-FITC (BD, cat.number 555837), and a monocyte panel with anti-CD14-FITC (Miltenyi, cat.number 130-110-518), anti-CD16-APC-Vio770 (Miltenyi, cat.number 130-096-655), anti-4-1BBL-APC (Miltenyi, cat.number 130-103-656), anti-Gal3-PE (Miltenyi, cat.number 130-101-315) and anti-Gal9-BV421 (Biolegend cat.number 348920). Both panels included a dump channel with anti-CD56-BV510 (BD, cat.number 659457) and viability-viogreen (Miltenyi, cat.number 130-110-206) (S1 Table). Following 10 minutes incubation in the dark at 4°C, cells were washed in flow running buffer and analysed using a MACS Quant Analyzer 10 (Miltenyi).

Data-files were analysed using FlowJo version 9 (Trestar inc., Ashland, OR). The lymphocyte gate was set on a forward-side scatter plot. T lymphocytes were identified as viability^-^CD56^-^CD4^+^CD8^-^, cytotoxic T lymphocytes as viability^-^CD56^-^CD4^-^CD8^+^ and classical monocytes as viability^-^CD56^-^CD14^+^CD16^-^(S1 and S2 Figs). CD3 was removed from the panel due to channel shortage after careful validation that this did not change the identification of CD4^+^ and CD8^+^ T lymphocytes (S3 Fig). The expression of receptors are reported as frequency of parent and median fluorescence intensity (MFI). Fluorescence minus five for T lymphocytes (excluding anti-4-1BB, anti-Gal9, anti-PD-1, anti-TIM3, and anti-OX40) and fluorescence minus three for monocytes (excluding anti-4-1BBL, anti-Gal3, and anti-Gal9) were validated against fluorescence minus one (FMO) and used throughout the flow cytometric experiments as controls.

### Intracellular staining

To assess cytokine production potentials, PBMCs were stimulated as described above and 50 ug/mL phorbol 12-myristate 13-acetate (PMA, Sigma-Aldrich) and 10 ug/mL Golgi-blocking agent brefeldin A (BFA, Sigma-Aldrich) was added for the last four hours of incubation. Additionally, half of the wells were also treated with 400 ng/mL recombinant humanised Galectin-9 (rhGal-9, R&D Solutions, cat.number 2045-GA). Cell permeabilization was performed as previously described [22] and staining performed with anti-IFN-γ-AF488 (Invitrogen, cat.number 53-7319-41) and anti-IL-10-PE-Cy7 (Invitrogen, 25-7108-41). Cells were incubated for 20 minutes room temperature before being washed twice to remove unbound antibodies. Isotype controls were included as controls. The gating strategy was optimized to the new panel (S4 Fig).

### Enzyme-linked immunosorbent assay

The levels of s4-1BB and Gal-9 in plasma were quantified using optimised, commercial s4-1BB and Gal-9 ELISA kits (Nordic Biosite, cat.number KBB-583 and R&D Systems, cat.number DGAL90) according to manufacturer’s instructions using a Thermo Scientific Multiskan GO [23]. Cut-off was defined as the lowest concentration where absorbance decreased linearly with concentration. The detection limit was 15.625 pg/mL for s4-1BB and concentrations below were assigned the value of the detection limit. For Gal-9, the detection limit was 0.078 pg/mL, and all measured values were above.

### Liver RNA sequencing

Liver expression of Gal-9 was measured using RNA sequencing on liver biopsies as previously described [24] and compared with pieces of healthy liver obtained during abdominal surgery.

### Statistics

Data were tested for normality using QQ-plots and histograms. A T test was used for comparison between two groups when data was normal distributed. Otherwise, log-transformation was attempted and when normal distribution was not achieved by log-transformation, a Mann-Whitney rank sum test was used for comparison between two groups. Association analyses were performed using the Spearman’s rank correlation test. Statistical analyses were performed using STATA (version IC 16.0, StataCorp). The results are expressed as median and interquartile range. A two-tailed p-value < 0.05 was considered statistically significant.

## Results

### Patient characteristics

Clinical and biochemical characteristics of the patients with alcoholic hepatitis (n = 35) are presented in Table 1. In this cohort, 8 of the 35 patients had died by day 90. We obtained follow-up samples from 24 patients at day 7 and 13 patients at day 90. At day 7, two patients (8.3%) had developed an infection. Infections were observed during follow-up in all patients dead by day 90.

**Table 1:** Characteristics.

	Patients			Healthy controls
	Day 0	Day 7	Day 90	
**Characteristics**				
	n = 35	n = 24	n = 13	n = 9
Gender (male/female)	21/14	17/7	7/6	7/2
Age (years)	55 [47–64]	56 [46.5–64]	56 [54–64]	52 [33–58]
ALT (U/I)	43 [31–71]	82 [48–105]	32.5 [22.5–50.5]	
Bilirubin (μmol/L)	193 [124–376]	164 [95–328]	15.5 [6.5–35]	
INR	1.8 [1.4–2.0]	1.6 [1.2–1.8]	1.25 [1.05–1.4]	
Albumin (g/L)	22 [19–25]	24 [21–28]	35 [30.5–39.5]	
Urea (mmol/L)	4.5 [2.5–10]	5.6 [3.95–19.8]	3.7 [2.95–7.7]	
Creatinine (μmol/L)	70 [53–104]	71 [57.5–109]	69 [62.5–77.5]	
C-reactive protein (mg/L)	22 [9.5–43.8]	18.6 [8.4–30.4]	4.8 [1.1–6.4]	
Leukocytes (10^9^/L)	11.3 [7.9–15.6]	12.95 [8.03–17.6]	6.92 [5.14–7.9]	
Neutrophils (10^9^/L)	8.1 [3.3–13.3]	8.54 [6.58–5.8]	3.39 [1.98–5.2]	
Lymphocytes (10^9^/L)	1.31 [1.02–1.89]	1.16 [0.95–1.4]	2.14 [1.37–2.78]	
*Complications*:				
Ascites (%)	54.3	41.7	8.33	
HE (%)	20.0	16.7	8.33	
Infection (%)	42.9	8.33		
*Disease severity*:				
GAHS	9 [7–10]	8 [7.5–9.5]	6 [6–7]	
MELD	21.5 [18–27]	19 [14–26]	8 [3.5–12]	
Child Pugh Score	10 [9–11]	9 [9–11]	6 [5–6]	
Child Pugh category (A/B/C)	(0/8/27)			
*Treatment*:				
Prednisolone/ Pentoxyfylline/None	16/2/17			

Clinical and biochemical baseline characteristics of the cohort of alcoholic hepatitis patients at diagnosis. ALT: Alanine aminotransferase. INR: International normalized ratio. GAHS: Glasgow alcoholic hepatitis score. HE: hepatic encephalopathy. MELD: Model of end-stage liver disease.

### Enhanced 4-1BB response of T lymphocytes

**Fig 1:**
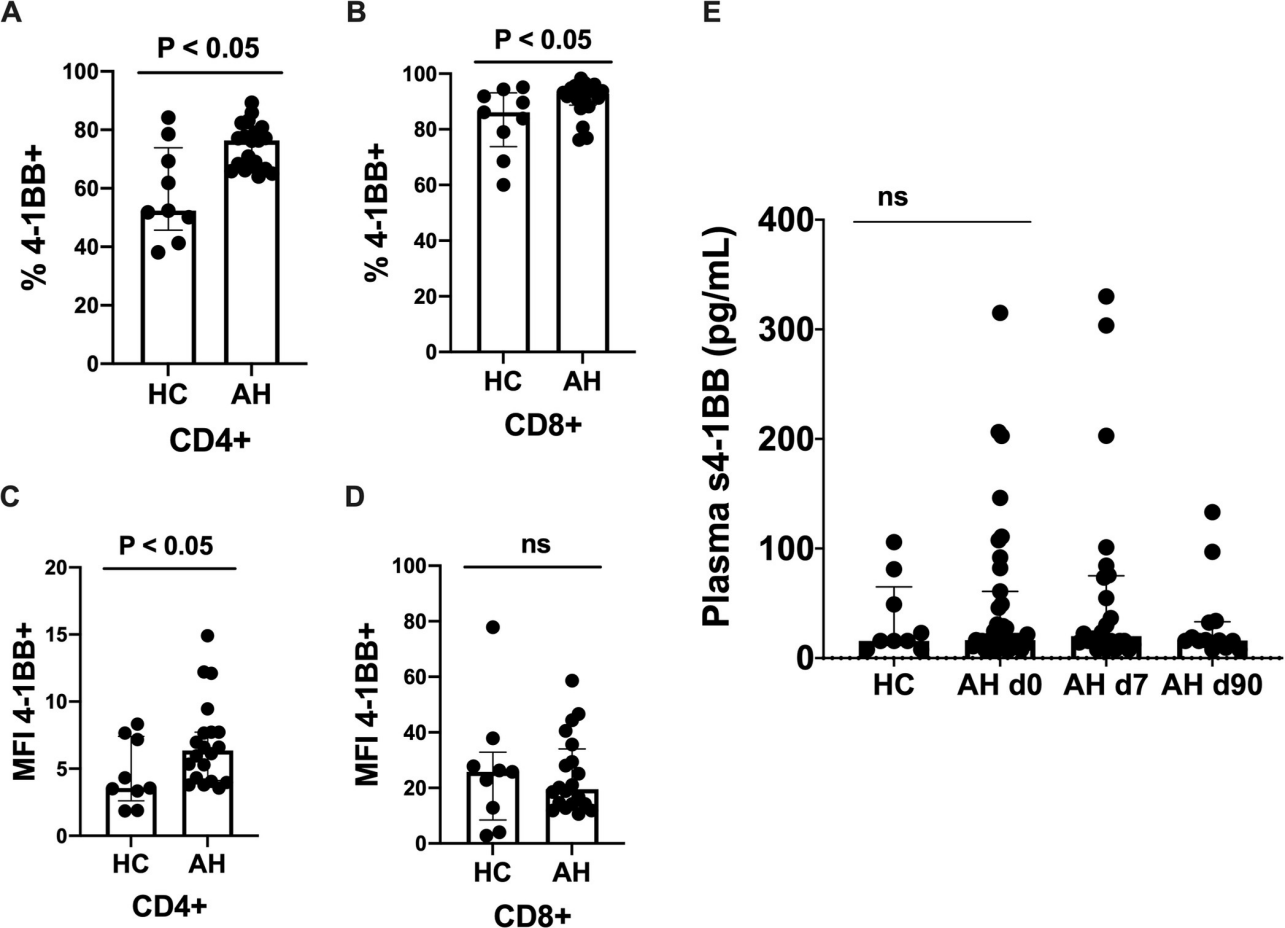
High expression of 4-1BB on T lymphocytes. Peripheral blood mononuclear cells were stimulated for 48 hours with anti-CD3 and anti-CD28. The frequency of (A) 4-1BB^+^ CD4^+^ and (B) CD8^+^ T lymphocytes are measured by flow cytometry and compared between patients with alcoholic hepatitis (AH) at diagnosis and healthy controls (HC). The median fluorescence intensity (MFI) of (C) 4-1BB^+^ CD4^+^ and (D) CD8^+^ T lymphocytes are compared between AH patients and HC. (E) Plasma soluble (s) 4-1BB was measured with enzyme-linked immunosorbent assay (ELISA) in HC and AH at diagnosis (day 0), day 7, and day 90. Median ± interquartile range shown. T test.

At diagnosis of AH, the frequencies of 4-1BB^+^ CD4^+^ and CD8^+^ T lymphocytes were increased compared with HC following anti-CD3 and anti-CD28 stimulation (Fig 1A and 1B). The spontaneous expression of 4-1BB was not elevated on CD4^+^ T lymphocytes but slightly increased on CD8^+^ T lymphocytes (S5 Fig). Furthermore, the MFI of 4-1BB was increased on the 4-1BB^+^ CD4^+^ T lymphocytes in the patients with AH compared with the HC, and this was not the case for CD8^+^ T lymphocytes (Fig 1C and 1D). In contrast, the frequencies of T lymphocytes expressing the co-stimulatory receptor OX40 following stimulation was similar in the patients with AH at diagnosis and the HC (S6A Fig). Plasma levels of s4-1BB remained stable from diagnosis to day 90 and were not significantly different from the HC (Fig 1E). Further, the frequency of PD-1^+^ CD4^+^ T lymphocytes were increased in AH as previously shown, but the frequency of TIM-3^+^ CD4^+^ T lymphocyte were similar between the groups (S6B and S6C Fig).

### Lower production of IL-10 in 4-1BB^+^ T lymphocytes

**Fig 2:**
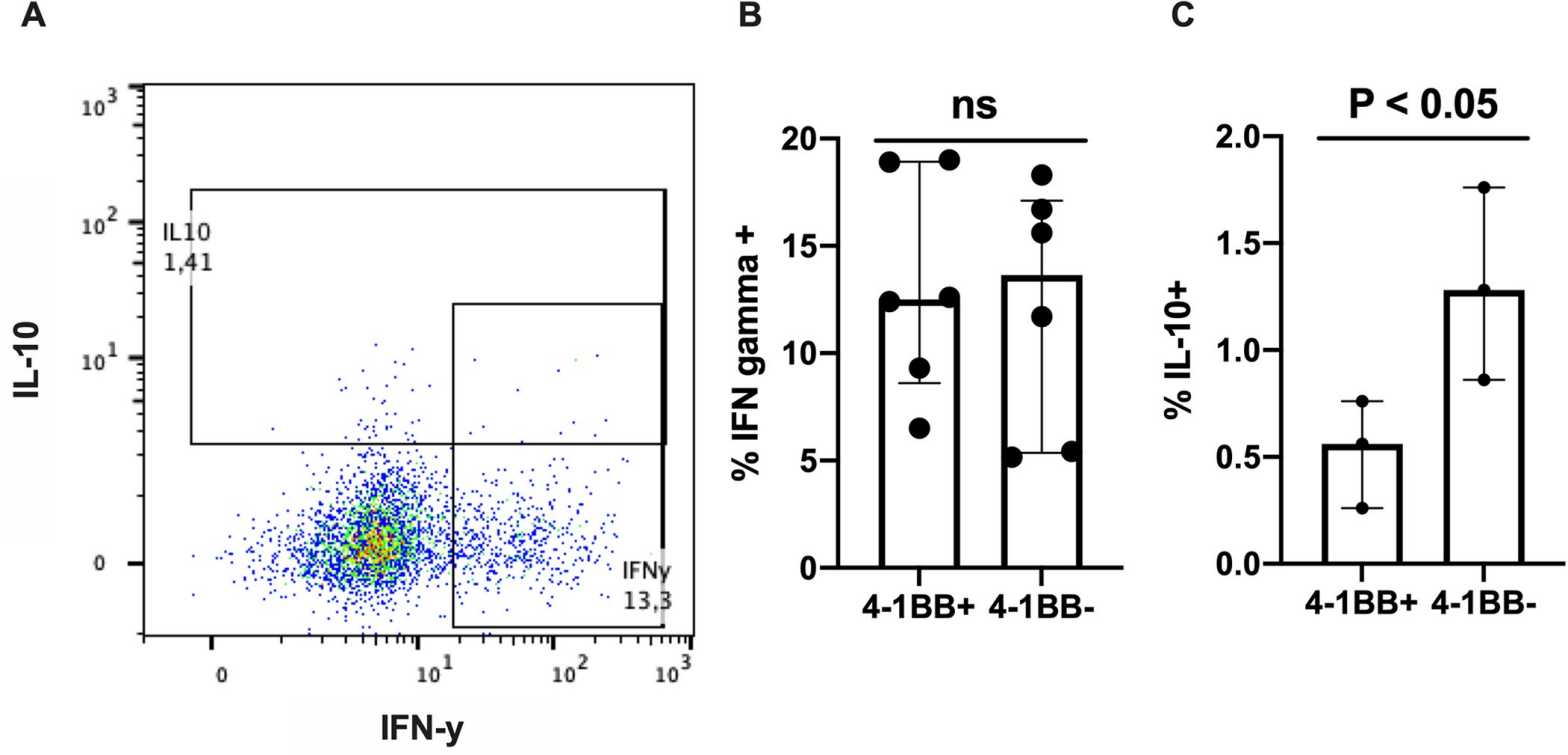
Cytokine production in 4-1BB^+^ and 4-1BB^-^ CD4^+^ T lymphocytes. Flow cytometry of peripheral blood mononuclear cells stimulated for 48 hours with anti-CD3 and anti-CD28 given PMA and brefaldin-A for the last 4 hours. (A) Typical flow cytometry plot of 4-1BB^+^ CD4^+^ T lymphocytes from a patient with alcoholic hepatitis showing interferon-γ (IFNy) on x-axis and interleukin-10 (IL-10) on y-axis. Frequencies of interferon-γ^+^ (B, n = 6) and IL-10^+^ (C, n = 3)) 4-1BB^+^ and 4-1BB^-^ CD4^+^ T lymphocytes in patients with alcoholic hepatitis. Differences between groups compared using paried T test. Graphs shown as median with interquartile range.

To explore the functional role of 4-1BB, we measured the expression of IFN-γ and IL-10 in T lymphocytes from patients with AH. The 4-1BB^+^ CD4^+^ T lymphocytes produced both IFN-γ and IL-10. The frequency of IFN-γ ^+^ T lymphocytes were not different when comparing 4-1BB^+^ with the 4-1BB^-^ T lymphocytes, but the frequency of IL-10-producing cells were lower in the 4-1BB^+^ T lymphocyte population (Fig 2A–2C).

### 4-1BB expression is associated with IFN-γ production during high PD-1 expression

**Fig 3:**
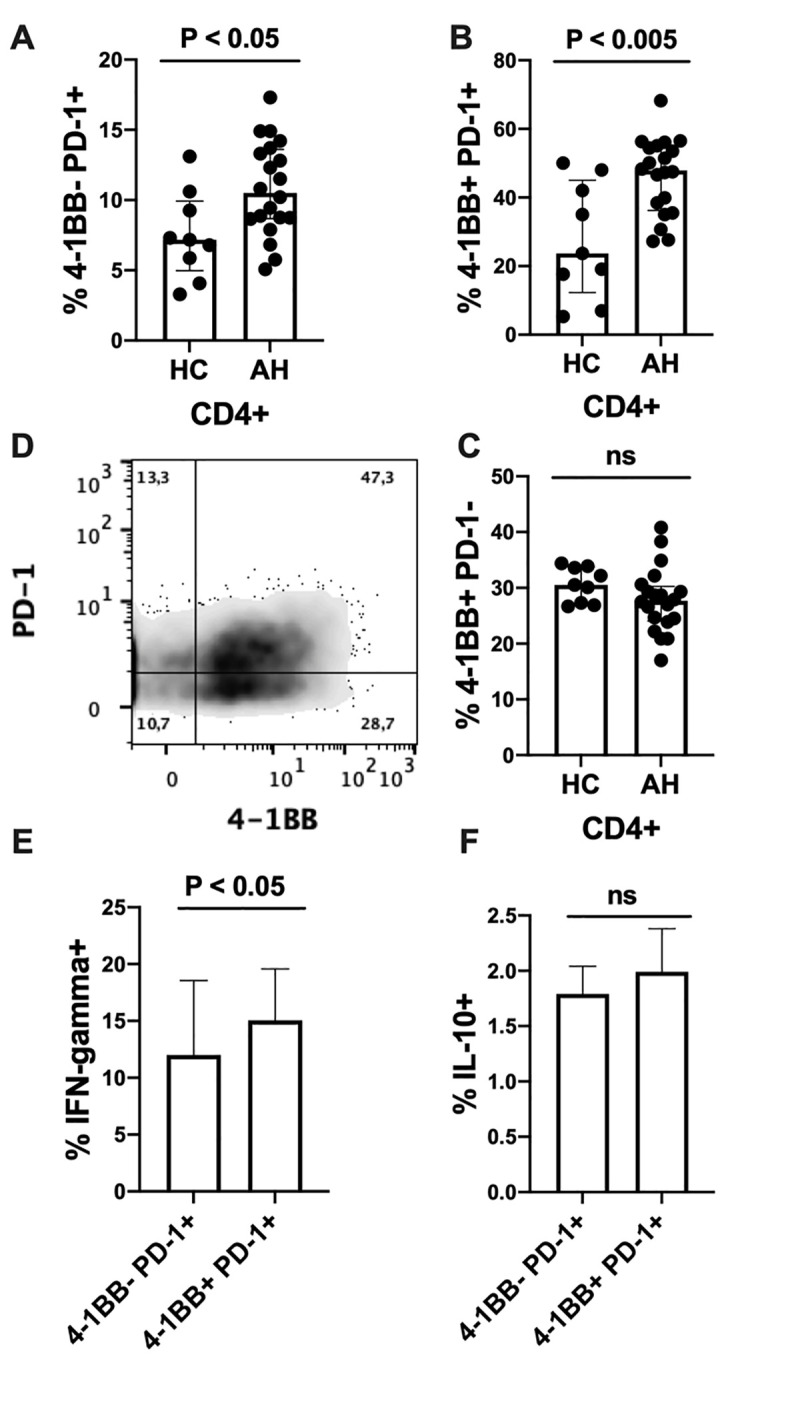
4-1BB in relation to PD-1 expression. Flow cytometry of peripheral blood mononuclear cells stimulated for 48 hours with anti-CD3 and anti-CD28. (A-C) CD4^+^ T Lymphocyte were co-stained for 4-1BB and Programmed Death receptor (PD)-1 and frequencies of subgroups are reported for healthy controls (HC) and patients with alcoholic hepatitis (AH) at the day of diagnosis. The frequencies of (E) interferon-γ (IFN-γ) and (F) interleukin-10 (IL-10) in the subgroups. All data analysed for differences between groups using unpaired (A-C) and paired (E-F) T test. All graphs shown as median ± interquartile range.

The frequencies of both 4-1BB^+^ PD-1^+^ CD4^+^ T lymphocytes and 4-1BB^-^ PD-1^+^ CD4^+^ T lymphocytes were increased in the patients with AH compared with the HC, whereas the frequencies of 4-1BB^+^ PD-1^-^ CD4^+^ T lymphocytes were similar between the groups following anti-CD3 and anti-CD28 stimulation (Fig 3A–3D). The frequency of IFN-γ^+^ cells were higher in the 4-1BB^+^ PD-1^+^ group compared with the 4-1BB^-^ PD-1^+^ group (Fig 3E). The subgroups showed no difference in frequency of IL-10^+^ cells (Fig 3F).

### Early changes in 4-1BB is associated with mortality

**Fig 4:**
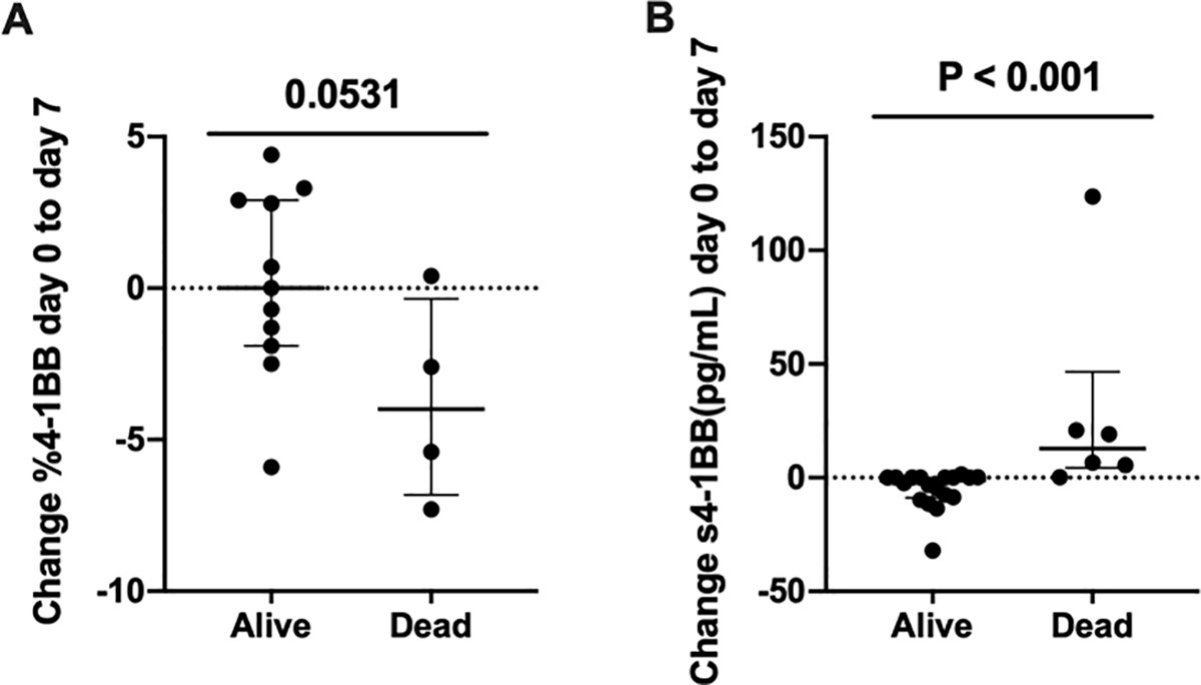
Early changes in 4-1BB is associated with mortality. Change in frequencies of 4-1BB^+^ CD4^+^ T lymphocytes measured using flow cytometry (A) and plasma s4-1BB measured with enzyme-linked immunosorbent assay (B) from day 0 to day 7 in patients with alcoholic hepatitis. Patients dead by day 90 compared with those still alive. Flow cytometry data compared using T test and ELISA data compared using Mann-Whitney rank sum test. Graphs shown as median ± interquartile range.

A decrease in 4-1BB^+^ CD4^+^ T lymphocytes from diagnosis to day 7 was associated with increased mortality by day 90 (Fig 4A). Furthermore, the patients who had died by day 90 had an increase in s4-1BB from day 0 to day 7 (Fig 4B). Two patients with AH developed infections by day 7 and they similarly had an early decrease in 4-1BB^+^ CD4^+^ T lymphocytes and increase in s4-1BB (S7 Fig). Neither the frequency of 4-1BB^+^ T lymphocytes nor baseline plasma s4-1BB correlated with disease severity scores. The frequency of 4-1BB^+^ CD8^+^ T lymphocytes was not associated with infection or mortality. Also, the association with clinical endpoints was not seen for PD-1 and OX40 (S8 Fig).

### Increased Gal-9 in alcoholic hepatitis

**Fig 5:**
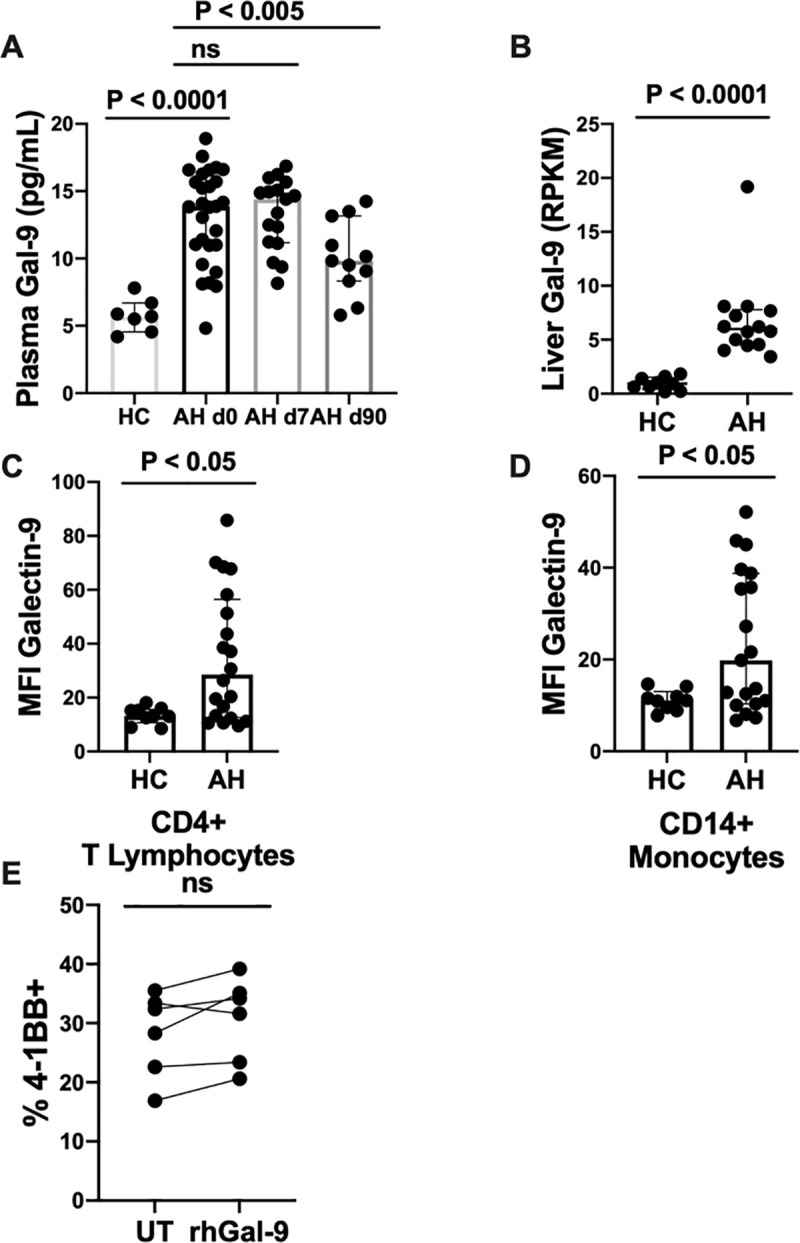
Galectin-9 expression and in vitro modulation. (A)Plasma, (B) liver, and (C-D) receptor levels of Galectin-9 (Gal-9) in healthy controls (HC) compared with alcoholic hepatitis patients (AH). (A) Plasma level of Gal-9 measured with enzyme-linked immunosorbent assay in healthy controls (HC), and alcoholic hepatitis patients (AH) day 0, day 7 and day 90. T test was used to compare HC and AH day 0. Paired T test was used on AH. (B) mRNA levels of Gal-9 in liver biopsies from HC and AH expressed as reads per kilobase million (RPKM) and compared using Wilcoxon signed-rank test. (C-D) Median fluorescence intensity (MFI) of Gal-9 on CD4^+^ T lymphocytes (C) and CD14^+^ monocytes (D) from flow cytometry analysis of peripheral blood mononuclear cells (PBMC). HC and AH compared using Mann-Whitney rank sum test. All shown as median with interquartile range. E: Flow cytometry analysis of PBMCs cultured for 4 hours untreated (UT) or in the presence of recombinant humanised Gal-9 (rhGal9) after 44 hours of pre-stimulation with anti-CD3 and anti-CD28.

In the patients with AH, plasma Gal-9 was increased compared with the HC (Fig 5A). Gal-9 remained elevated by day 7 after diagnosis in the patients with AH, but were lower at day 90 compared to the day of diagnosis. Furthermore, the liver showed increased amounts of Gal-9 mRNA in the patients with AH compared with the HC (Fig 5B). Increased Gal-9 MFI was also found on both CD4^+^ T (viability > 95%) lymphocytes, and monocytes (Fig 5C and 5D). Treatment of PBMCs with rhGal-9 was non-toxic and binding of rhGal-9 was visualised as a dose-dependent increase in MFI of Gal-9. However, the frequency of 4-1BB^+^ CD4^+^ T lymphocytes was not significantly changed by rhGal-9 treatment (Fig 5E).

## Discussion

In this study, we report an enhanced stimulated 4-1BB response in T lymphocytes in patients with AH. Early loss of 4-1BB expressing T lymphocytes is associated with increased short-term mortality, and addition of extracellular Gal-9 seems not to be sufficient to reverse this.

Our study is exploratory and descriptive by nature and functional assays are employed *ex vivo*. This may be a limitation as the used stimulation activates T lymphocytes but does not reflect the *in vivo* response towards infections. Furthermore, this study mainly focused on T lymphocytes despite the *in vivo* immune response being a complex interplay between multiple immunological factors. As the disease is rare in Denmark, the cohort is relatively small and few patients meet a clinical endpoint resulting in only two patients having an infection at day 7 in our cohort. This imposes limitations on the degrees of detail with which these findings can be interpreted. Further, some of the patients received immunomodulatory drugs, which potentially can change the expression of T lymphocyte receptors. However, we did not find any association between the use of immunomodulatory drugs and receptor expression, but cannot rule out an impact of these drugs.

The increased stimulated 4-1BB response in AH is consistent with that of patients with rheumatoid arthritis [14]. Also, the levels of s4-1BB in plasma were comparable with the levels found in studies of other inflammatory diseases [14, 25] and was not different from concentrations in healthy controls as recently published [26]. AH is characterized by an overt systemic inflammatory response in combination with vast liver inflammation. This is proposed to be intimately linked to a compensatory immunosuppressive phase further increasing these patients’ susceptibility to infections in line with knowledge from sepsis [27]. Our data supports this phenomenon as T lymphocytes have high expression of PD-1, but we add new insights into the complexity of T lymphocyte activation by showing that patients with AH have a preserved and increased ability to express 4-1BB in response to stimulation maybe to overcome the increased expression of inhibitory receptors. In our patients, we found an increased expression of PD-1 as previously described [17], but we found only low expressions close to undetectable of TIM-3 without difference between the patients and HC nor did we observe an association between mortality and the proportion of inhibitory receptor positive T lymphocytes.

We show that expression of 4-1BB is related to higher production of IFN-γ and lower production of IL-10. Increased IFN-γ production in AH is found in the most activated T lymphocytes co-expressing 4-1BB and PD-1, as PD-1 is expressed transiently after activation [28, 29]. IFN-γ is a key anti-bacterial cytokine, which amongst other functions promote activation of macrophages and natural killer cells, which thereby may link 4-1BB to antimicrobial functions. The decreased IL-10 production in 4-1BB^+^ T lymphocytes may again support that 4-1BB expression reflects activated T lymphocytes also in alcoholic hepatitis. These data are, however, based on ex vivo experiments, and we do not know to what extent this reflect in vivo functions.

Our findings are of clinical relevance as an early decrease in 4-1BB^+^ T lymphocytes is associated with increased mortality. This may mechanistically be explained by enhanced IFN-γ production of the 4-1BB^+^ T lymphocytes as indicated by our *in vitro* data. In support, our data point to infections being a reason for the association between 4-1BB and mortality, but this needs to be verified in a larger cohort. It is, however, shown experimentally that 4-1BB^-/-^ knockout mice have decreased clearing of certain bacteria e.g. *Staphylococcus Aureus* [11, 12]. The reason for the loss of 4-1BB expressing lymphocytes, whether this is a cause or a consequence of infections remain to be explored.

Soluble 4-1BB was related to the development of infection and short-term mortality in a manner exactly opposite to that of T lymphocyte 4-1BB even though no negative correlation between T lymphocyte 4-1BB and s4-1BB was observed in our cohort. The functional role and clinical relevance of s4-1BB remains largely unknown. However, an increase in s4-1BB may enhance the inhibition of the bidirectional signaling between 4-1BB and its cognate ligand, 4-1BBL, expressed on antigen presenting cells (APCs) [16, 30]. Thus, a decreased expression and an increased s4-1BB may work synergistically to lower T lymphocyte and monocyte antimicrobial functions which contribute to an increased susceptibility to infection contributing to death [31, 32].

In light of this, it may be desirable to cautiously boost 4-1BB signalling in AH. Gal-9 was an obvious candidate to attempt to balance co-stimulatory and inhibitory receptor expression, as Gal-9 can bind both 4-1BB and the inhibitory TIM-3 receptor and modulate receptor signaling [14, 18, 19]. We found a high presence of Gal-9 on the surface of T lymphocytes in AH as also previously indicated [17] and extended these observations to include monocytes, liver and blood, where Gal-9 was also elevated. In vitro, however, we saw no change in T lymphocyte receptor expression or cytokine production by addition of exogenous Gal-9 during neither TCR-targeted stimulation with anti-CD3 and anti-CD28 nor the monocyte-targeted stimulation with LPS. This may be explained by the dual binding to both 4-1BB and TIM-3 in a way that merely sustains the already present expression. Additionally, the high levels of Gal-9 in vivo may already have modulated the T lymphocytes in a way that no further functional modulation could be detected, although we did see an ability of the T lymphocytes to bind additional Gal-9.

In conclusion, stimulated T lymphocyte 4-1BB response is increased in patients with AH and loss of 4-1BB is associated with higher short-term mortality. This suggests a role of 4-1BB on T lymphocyte activation in alcoholic hepatitis.

## Supporting information

S1 Table Antibodies used for flow cytometry. (DOCX)

S1 Fig Gating strategy for T lymphocytes. Flow cytometry analysis of peripheral blood mononuclear cells (PBMCs) stimulated for 48 hours with anti-CD3 and anti-CD28. Lymphocytes were identified on a forward scatter (FSC) versus side scatter (SSC) plot. Duplets were excluded. Alive lymphocytes were identified as CD56^-^Viability^-^. CD4^-^CD8^-^ cells were excluded. CD4^+^ and CD8^+^ T lymphocytes were identified and their receptor expressions were based on flourescence-minus-5 controls.
(TIF)

S2 Fig Gating strategy for monocytes. Flow cytometry analysis of peripheral blood mononuclear cells (PBMCs). Monocytes were identified on a forward scatter (FSC) versus side scatter (SSC) plot. Duplets were excluded. Alive monocytes were identified as CD56^-^Viability^-^. Classical monocytes were identified as CD14^+^CD16^-^ and receptor expression of galectin-9 (Gal9) were based on fluorescence-minus-three controls.
(TIF)

S3 Fig Gating with and without anti-CD3. Flow cytometry analysis of peripheral blood mononuclear cells comparing the gating strategy (A) with and (B) without anti-CD3.
(TIF)

S4 Fig Identification of T lymphocytes after stimulation. Flow cytometry identification of (A) CD4^+^ and (B) CD8^+^ T lymphocytes from peripheral blood mononuclear cells after 48 hours stimulation with anti-CD3 and anti-CD28 and 4 hours with phorbol 12-myristate 13-acetate (PMA) and Golgi-blocking agent brefeldin A (BFA).
(TIF)

S5 Fig Spontaneous 4-1BB expression on T lymphocytes. Peripheral blood mononuclear cells were analyzed using flow cytometry and the frequency of (A) 4-1BB^+^ CD4^+^ and (B) CD8^+^ T lymphocytes were compared between patients with alcoholic hepatitis (AH) at diagnosis and healthy controls (HC). Median ± interquartile range shown. T test.
(TIF)

S6 Fig Co-receptor expression on CD4^+^ T lymphocytes. Flow cytometry analysis of peripheral blood mononuclear cells (PBMCs) stimulated for 48 hours with anti-CD3 and anti-CD28. Frequencies of CD4+ T Lymphocytes expressing (A) OX40, (B) PD-1, and (C) TIM-3 in healthy controls (HC) compared with alcoholic hepatitis patients (AH). T test used for comparisons between groups. Graph shown as median with interquartile range.
(TIF)

S7 Fig Early changes in 4-1BB is associated with infection. Change in frequencies of 4-1BB^+^ CD4^+^ T lymphocytes measured using flow cytometry (A) and plasma s4-1BB measured with enzyme-linked immunosorbent assay (B) from day 0 to day 7 in patients with alcoholic hepatitis. Patients infected at day 7 (n = 2) compared with patients without infection. Flow cytometry data compared using T test and ELISA data compared using Mann-Whitney rank sum test. Graphs shown as median ± interquartile range.
(TIF)

S8 Fig Early change in PD-1 and OX40 is not associated with mortality. Change in frequencies of (A) PD-1^+^ and (B) OX40^+^ CD4^+^ T lymphocytes from day 0 to day 7 measured using flow cytometry. Patients dead by day 90 (dead, n = 4) compared with those still alive using T test. Graph shown as median ± interquartile range.
(TIF)

## List of abbreviations

ADAM: a disintergrin and metalloprotease-17
AH: alcoholic hepatitis
ALT: alanine transferase
APC: antigen-presenting cell
DMSO: dimethyl sulfoxide
ELISA: enzyme-linked immunosorbent assay
FMO: fluorescence minus one
GAHS: Glasgow alcoholic hepatitis score
Gal: galectin
HC: healthy controls
HE: hepatic encephalopathy
IFN: interferon
IL: Interleukin
INR: international normalized ratio
L: ligand
LPS: lipopolysaccharide
MELD: Model of end-stage liver disease
MFI: median fluorescence intensity
PBMCs: Peripheral blood mononuclear cells
PBS: phosphate-buffered saline
PD-1: programmed death receptor 1
PMA: phorbol 12-myristate 13-acetate
rhGal-9: recombinant humanized Galectin-9
RNA: ribonucleic acid
RPMI: Roswell Park Memorial Institute
s: soluble
TCR: T cell receptor
TIM-3: T cell immunoglobulin mucin-3
TNF-α: tumor necrosis factor-alpha
