# Supplementary material for: Early loss of T lymphocyte 4-1BB receptor expression is associated with higher short-term mortality in alcoholic hepatitis
Source: PLoS One. 2021 Aug 5;16(8):e0255574. doi: 10.1371/journal.pone.0255574 (PMC8341529; doi:10.1371/journal.pone.0255574)
Supplement: S1 Table — (DOCX) [file pone.0255574.s001.docx]

| S1 Table: Antibodies used for flow cytometry | | | | |
| --- | --- | --- | --- | --- |
| **Antigen** | **Flourochrome** | **Clone** | **Manufacturer** | **Cat.nr.** |
| CD4 | PerCP-Vio700 | Rea623 | Miltenyi | 130-113-228 |
| CD8 | Pe-Vio770 | Rea734 | Miltenyi | 130-110-680 |
| 4-1BB | APC | 4B4-1 | Biolegend | 309810 |
| Gal9 | BV421 | 9M1-3 | Biolegend | 348920 |
| PD-1 | PE | EH12.2H7 | Biolegend | 329906 |
| TIM3 | eFlour-780 | F38-2E2 | eBioscience | 47-3109-42 |
| OX40 | FITC | MOPC-21 | BD | 555837 |
| CD14 | FITC | Rea599 | Miltenyi | 130-110-518 |
| CD16 | APC-Vio770 | VEP13 | Miltenyi | 130-096-655 |
| 4-1BBL | APC | Rea255 | Miltenyi | 130-103-656 |
| Gal3 | PE | M3/38 | Miltenyi | 130-101-315 |
| CD56 | BV510 | NCAM16.2 | BD | 659457 |
| Viability | Viogreen |  | Miltenyi | 130-110-206 |
| IFN-γ | AF488 | 4S-B3 | Invitrogen | 53-7319-41 |
| IL-10 | PE-Cy7 | JES-9D7 | Invitrogen | 25-7108-41 |
| Characteristics of the antibdoies used for the flow cyteometrics analyses | | | | |
