# Supplementary figures and images for: Early loss of T lymphocyte 4-1BB receptor expression is associated with higher short-term mortality in alcoholic hepatitis
Source: PLoS One. 2021 Aug 5;16(8):e0255574. doi: 10.1371/journal.pone.0255574 (PMC8341529; doi:10.1371/journal.pone.0255574)

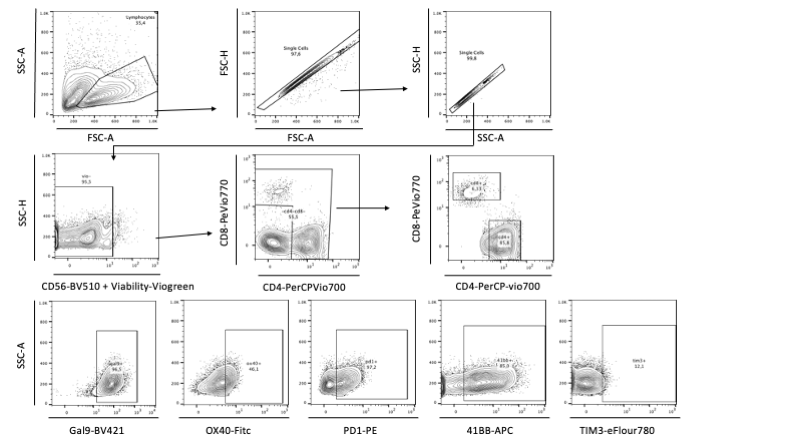

Supplement: S1 Fig — Flow cytometry analysis of peripheral blood mononuclear cells (PBMCs) stimulated for 48 hours with anti-CD3 and anti-CD28. Lymphocytes were identified on a forward scatter (FSC) versus side scatter (SSC) plot. Duplets were excluded. Alive lymphocytes were identified as CD56-Viability-. CD4-CD8- cells were excluded. CD4+ and CD8+ T lymphocytes were identified and their receptor expressions were based on flourescence-minus-5 controls. (TIF) [file pone.0255574.s002.tif]

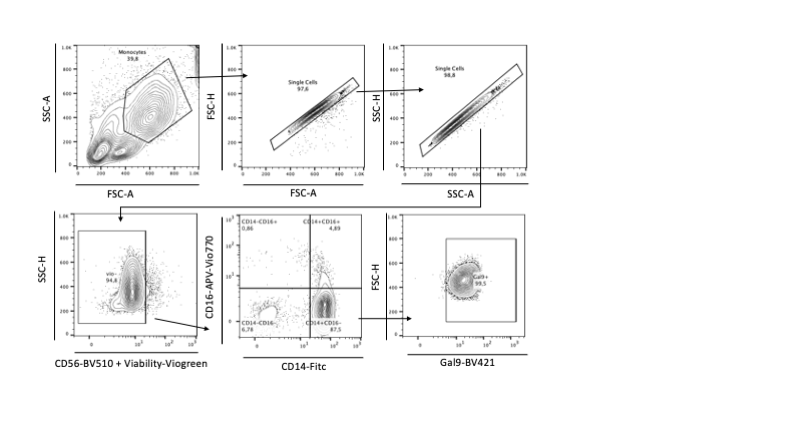

Supplement: S2 Fig — Flow cytometry analysis of peripheral blood mononuclear cells (PBMCs). Monocytes were identified on a forward scatter (FSC) versus side scatter (SSC) plot. Duplets were excluded. Alive monocytes were identified as CD56-Viability-. Classical monocytes were identified as CD14+CD16- and receptor expression of galectin-9 (Gal9) were based on fluorescence-minus-three controls. (TIF) [file pone.0255574.s003.tif]

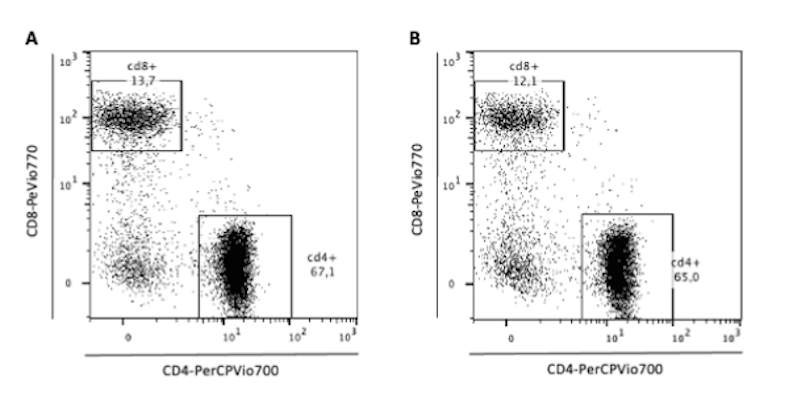

Supplement: S3 Fig — Flow cytometry analysis of peripheral blood mononuclear cells comparing the gating strategy (A) with and (B) without anti-CD3. (TIF) [file pone.0255574.s004.tif]

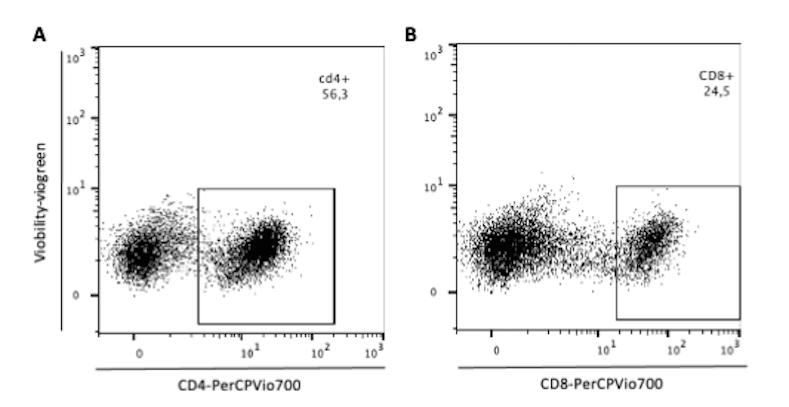

Supplement: S4 Fig — Flow cytometry identification of (A) CD4+ and (B) CD8+ T lymphocytes from peripheral blood mononuclear cells after 48 hours stimulation with anti-CD3 and anti-CD28 and 4 hours with phorbol 12-myristate 13-acetate (PMA) and Golgi-blocking agent brefeldin A (BFA). (TIF) [file pone.0255574.s005.tif]

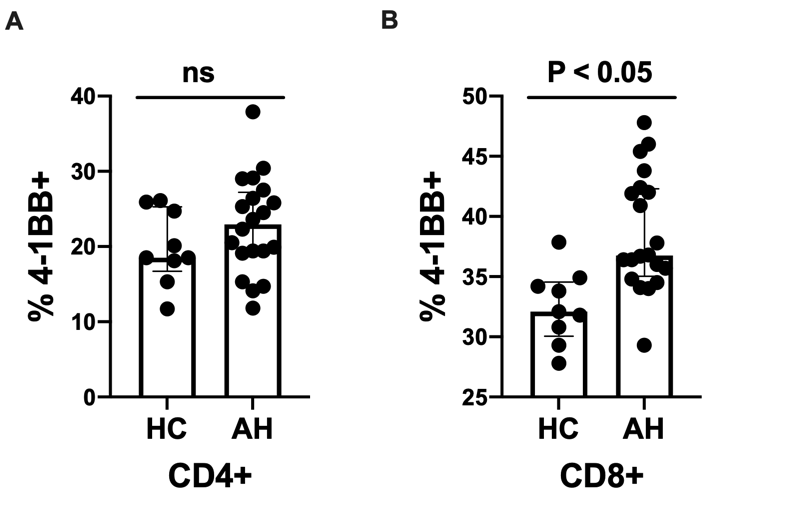

Supplement: S5 Fig — Peripheral blood mononuclear cells were analyzed using flow cytometry and the frequency of (A) 4-1BB+ CD4+ and (B) CD8+ T lymphocytes were compared between patients with alcoholic hepatitis (AH) at diagnosis and healthy controls (HC). Median ± interquartile range shown. T test. (TIF) [file pone.0255574.s006.tif]

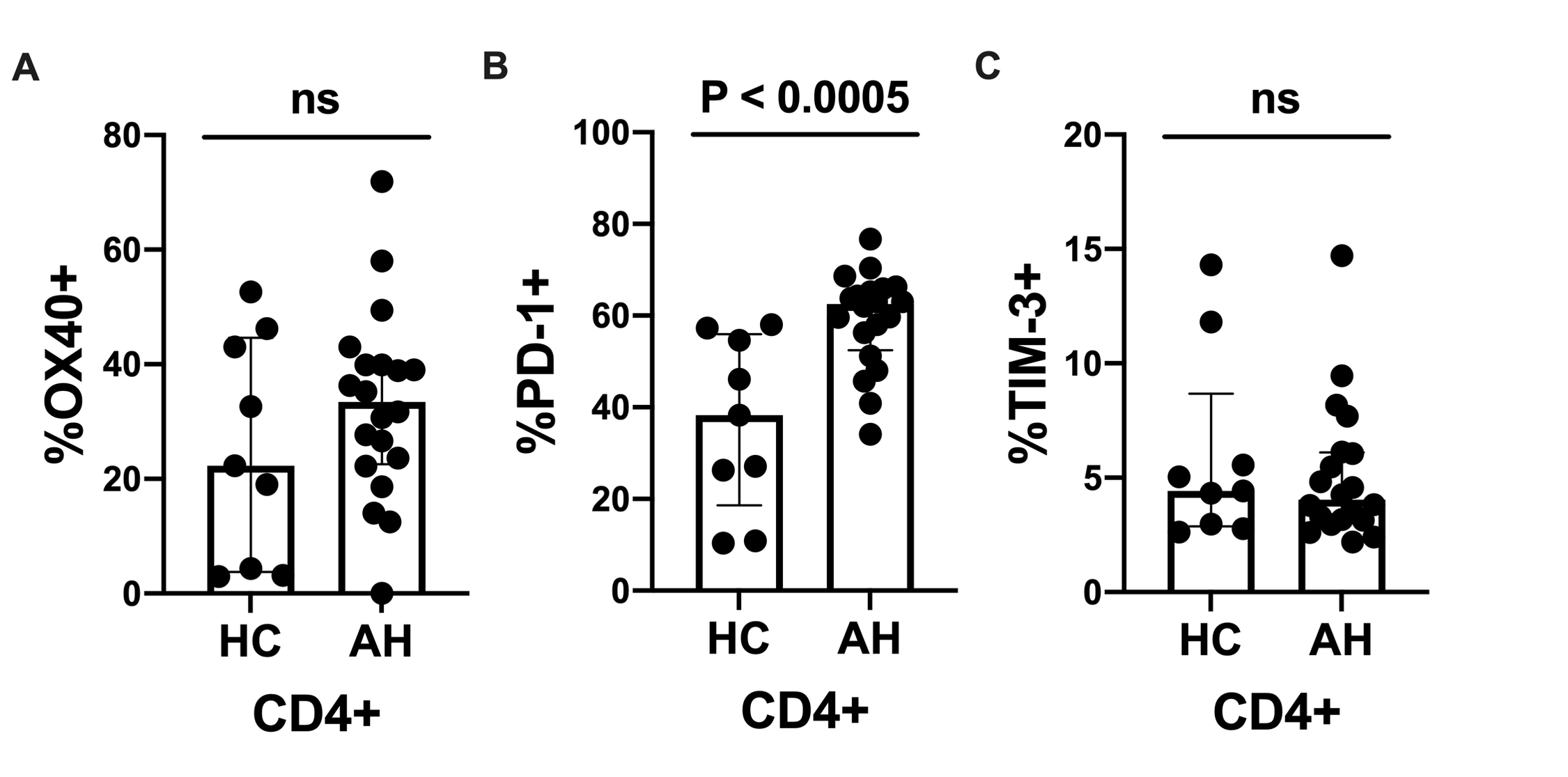

Supplement: S6 Fig — Flow cytometry analysis of peripheral blood mononuclear cells (PBMCs) stimulated for 48 hours with anti-CD3 and anti-CD28. Frequencies of CD4+ T Lymphocytes expressing (A) OX40, (B) PD-1, and (C) TIM-3 in healthy controls (HC) compared with alcoholic hepatitis patients (AH). T test used for comparisons between groups. Graph shown as median with interquartile range. (TIF) [file pone.0255574.s007.tif]

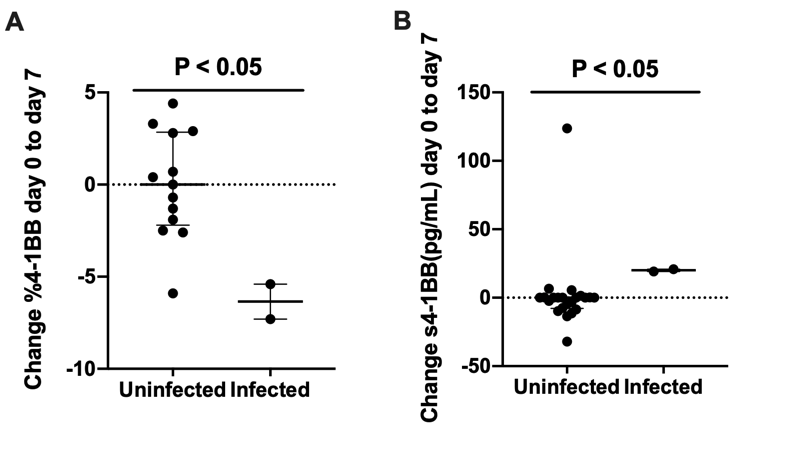

Supplement: S7 Fig — Change in frequencies of 4-1BB+ CD4+ T lymphocytes measured using flow cytometry (A) and plasma s4-1BB measured with enzyme-linked immunosorbent assay (B) from day 0 to day 7 in patients with alcoholic hepatitis. Patients infected at day 7 (n = 2) compared with patients without infection. Flow cytometry data compared using T test and ELISA data compared using Mann-Whitney rank sum test. Graphs shown as median ± interquartile range. (TIF) [file pone.0255574.s008.tif]

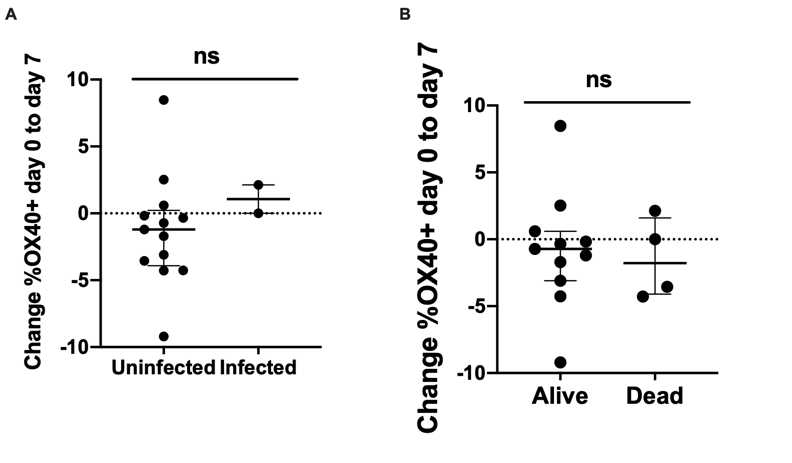

Supplement: S8 Fig — Change in frequencies of (A) PD-1+ and (B) OX40+ CD4+ T lymphocytes from day 0 to day 7 measured using flow cytometry. Patients dead by day 90 (dead, n = 4) compared with those still alive using T test. Graph shown as median ± interquartile range. (TIF) [file pone.0255574.s009.tif]
